# Oxytocin Attenuates Methamphetamine-Induced Apoptosis *via* Oxytocin Receptor in Rat Hippocampal Neurons

**DOI:** 10.3389/fphar.2021.639571

**Published:** 2021-08-12

**Authors:** Chunli Li, Haipeng Wang, Min Wang, Chunyun Chen, Fei Bai, Mengqi Ban, Chunfu Wu

**Affiliations:** Department of Pharmacology, Shenyang Pharmaceutical University, Shenyang, China

**Keywords:** methamphetamine, oxytocin, atosiban, oxytocin receptor, hippocampal neurons, apoptosis

## Abstract

Methamphetamine (METH) is a highly neurotoxic psychoactive substance that can directly damage the central nervous system through prolonged use. Oxytocin (OT) has attracted much attention because of its neuroprotective effect. The purpose of this study was to investigate whether OT is neuroprotective against METH-induced damage in rat hippocampal neurons. Our results revealed that pre-incubation with OT significantly prevented the damage of METH to hippocampal neurons, including the decrease of mitochondrial membrane potential and the increase of ROS (reactive oxygen species). OT pre-incubation attenuated the up-regulation of Cleaved-Caspase-3 expression and the down-regulation of Bcl-2/Bax expression induced by METH. Pre-incubation with OT prevented the decrease in oxytocin receptor density and P-CREB (phosphorylation of cAMP-response element binding) expression induced by METH in rat hippocampal neurons. Moreover, Pre-incubation of atosiban (ATO) significantly prevented these changes. In conclusion, our study proved that pre-administration of OT could significantly attenuate hippocampal neuron apoptosis induced by METH. Oxytocin receptor activation is involved in the preventive effect of OT on METH-induced apoptosis in rat hippocampal neurons.

## Introduction

The expanding abuse of METH has become a serious public crisis because of its strong addictiveness. Excessive overuse of METH will cause various mental symptoms and also be harmful to the central nervous system. METH is a cationic lipophilic molecule that easily enters and stores in mitochondria, reduces mitochondrial membrane potential and interferes with the mitochondrial biosynthesis, such as ATP synthesis, resulting in increased levels of reactive oxygen species (ROS), ultimately leading to apoptosis (Lemasters et al., 1999; Wu et al., 2007). METH has been shown to cause cell death through dopa-mediated and glutamate-mediated pathways: After METH enters the dopaminergic matrix, it will promote the oxidation of dopamine to superoxide, and superoxide and nitric oxide react to form peroxynitrite, which leads to cell death; Dopamine can also promote the release of glutamate to activate NMDA receptor, resulting in increased production of nitric oxide and activation of the caspase cascade (Davidson et al., 2001). In addition, the ability of METH to penetrate the blood-brain barrier can make it easier to enter the brain to exert toxic effects (Srisurapanont et al., 2003; Shah and Kumar, 2016; Kish et al., 2017). However, the neurotoxic mechanism of METH is not completely understood. The possible mechanisms of METH-induced central nervous system injury include mitochondrial dysfunction, neuronal apoptosis, oxidative stress, and excitotoxicity (Murphy et al., 1999; Deng et al., 2002).

OT neurons are widely distributed in the central nervous system, and they perform as a central neurotransmitter or neuromodulator to participate in the regulation of reward, stress, learning, memory, social behavior and drug dependence (Smith et al., 2010; Churchland and Winkielman, 2012; Young, 2015). OT treatment has an inhibitory effect on METH primed reinstatement, while the vagus nerve and vasopressin V1A receptor mediate the inhibitory effect of OT on the self-administration and seeking of METH in rats (Everett et al., 2018; Everett et al., 2020; Everett et al., 2021). A trial showed that OT may safely improve treatment attendance of patients with METH use disorder (Stauffer et al., 2020). After treatment with OT, it was found that the activity of hippocampal neurons was significantly improved either it was detected immediately after oxygen deprivation or after reoxygenation for 6 h after oxygen deprivation (Ceanga et al., 2010). Repeated administration of OT can promote hippocampal cell proliferation and dendritic cell maturation (Sánchez-Vidaña et al., 2016). In addition, OT can reduce rotenone-induced apoptosis in striatum neurons, suggesting that OT can protect damaged neurons through anti-apoptotic pathways (Erbaş et al., 2012).

Both humans and rodents express oxytocin receptor (OTR) in the brain. There’re studies supporting that oxytocin receptor express much in the dorsal cortex, anterior olfactory nucleus, limbic system and hypothalamic nucleus of adult rats (Insel and Shapiro, 1992; Barberis et al., 2010; Kremarik et al., 2010; Tribollet et al., 2010). OT in the central nervous system can exert a variety of physiological functions through oxytocin receptor. OT can alter the effects of stress on synaptic plasticity and spatial memory in the rat hippocampus by oxytocin receptor (Tomizawa et al., 2003; Sun-Young et al., 2015). Previous studies have found that OT could significantly inhibit the formation of METH-induced conditioned place preference (CPP) and the recurrence of CPP caused by restraint stress. These effects can be prevented by atosiban (ATO), oxytocin receptor antagonist (Qi et al., 2009). Previous *in vitro* study indicated OT offered protectiveness in the condition of cerebral ischemia and oxygen-rich hippocampal neurons and all these could be prevented by ATO (Ceanga et al., 2010). Combined with all above, we consider that OT may have neuroprotective effects associated with oxytocin receptor.

## Materials and Methods

### Materials

METH was obtained from Criminal Investigation Institute of Liaoning Province. OT was obtained from Key Organics. Phosphate buffer saline (PBS) was obtained from Hyclone. Atosiban, Dimethyl sulfoxide (DMSO), 4′, 6-diamidino-2-phenylindole (DAPI), Poly-l-lysine and MTT were obtained from Sigma-Aldrich. B27 supplement, Neurobasal Medium, Fetal Bovine Serum (FBS), 0.25% Trypsin-EDTA, DMEM/F12 medium and penicillin/streptomycin were obtained from Gibco. Mitochondrial membrane potential assay kit with JC-1, ROS Assay Kit, BCA protein assay kit, Alexa Fluor 488, Goat Anti-Rabbit lgG and Goat Serum were obtained from Beyotime Biotechnology. Horseradish peroxidase (HRP)-goat anti-mouse IgG and HRP-goat anti-rabbit IgG were obtained from Nakasugi Golden Bridge. Anti-Oxytocin Receptor and anti-beta III Tubulin antibodies were obtained from Abcam. Bax, CREB, Anti-Phospho-CREB (P-CREB), Anti-P44/42MAPK (ERK1/2), Anti-Phospho-P44/42MAPK (P-ERK1/2), Anti-Caspase-3 and anti-Cleaved Caspase-3 antibodies were obtained from Cell Signaling Technology. Anti-Bcl-2 Polyclonal antibody was obtained from Proteintech. Anti-beta-actin antibody was obtained from Santa Cruz Biotechnology. Anti-GAPDH antibody was obtained from Hangzhou Xianzhi Biological Technology Co., Ltd.

### Animals and Culture of Primary Hippocampal Neurons

Male and female Sprague Dawley rats (250–280 g) were used to obtain neonatal rats were from Liaoning Changsheng Biotechnology Co., Ltd. Primary hippocampal neurons were prepared as previously described with minor modifications (Zhang et al., 2010). Briefly, hippocampal regions were removed from Sprague Dawley rats which were neonatal within 24 h in Ca^2+^ and Mg^2+^ free Hank’s balanced salt solution under a stereo microscope in a sterile environment. After the hippocampus tissue was cut into small pieces by ophthalmological scissors, an appropriate amount of 0.25% Trypsin-EDTA was added to the shredded hippocampus and then digested in a 37°C water bath for 15 min. Trypsin digestion was then stopped by adding an appropriate amount of DMEM/F12 medium containing 10% FBS and discard the supernatant. Add fresh DMEM/F12 containing 10% FBS culture medium and gently blow the hippocampus tissue into a single cell with a hot-polished plastic pipette. After standing for about 1 min, the supernatant cell suspension was filtered through a 200 mesh cell sieve to prepare a single cell suspension. Aspirate the mixed cell suspension for cell counting. The cell density was adjusted to 5×10^5^ cells/mL and inoculated into a culture plate coated with 0.01% poly-lysine in advance, gently mixed and cultured in a humidified 95% air, 5% CO_2_ incubator. After 4 h of culture, the culture medium was replaced with a medium containing 86% Neurobasal medium, 10% FBS, 2% B27, 1% glutamine stock solution, and 1% double antibody (penicillin, streptomycin). After that, half of the culture solution was replaced every 2 d, and neurons cultured for 7–10 d were selected for subsequent experiments. The rats were used in this study and the cultivation protocol of primary hippocampal neurons were reviewed by the Animal Ethics Committee of Shenyang Pharmaceutical University, and the regulations were consistent with the Guideline for the Care and Use of Laboratory Animals published by the National Institutes of Health.

### Purity Identification of Hippocampal Neurons

Neuron purity were identified as described previously (Zhang et al., 2010). Adding appropriate amount of 4% PFA fixed hippocampal neurons for 20 min. Then cells were permeabilized with 0.03% Triton X-100 for 10 min, block with 5% goat serum for 1 h at room temperature. Incubate with 0.1% beta-Tubulin III diluted in PBS at 4°C overnight and rinse with phosphate buffer saline with 0.1% Tween-20 (PBST). 0.5% Alexa Fluor 488-labeled goat anti-rabbit secondary antibody was added and incubated for 1 h at room temperature in the dark, then rinsed with an appropriate amount of PBST. Add DAPI staining in the dark for 10 min, discard the stain, and add a proper amount of PBST to rinse. After adding an appropriate amount of PBST to each well, the cells were observed using an inverted fluorescence microscope.

### Drug Treatment

The primary hippocampal neurons were then randomly divided into six groups. The control cells were cultured for 24 h in a medium without drug, and the cells in OT (0.01–1 μM) group, METH (1–8 mM) group and ATO (0.01–1 μM) group were cultured for 24 h in a drug-containing medium. OT + METH group and ATO + OT + METH group were pre-incubated with optimal concentration of OT (1 μM) and/or ATO (1 μM) for 2 h before treatment with METH, and then treated with METH (4 mM) for 24 h. After drug treatment, cells in each group were tested for cell viability by MTT assay. ROS, mitochondrial membrane potential, Cleaved Caspase-3 and p-ERK1/2 were detected by immunofluorescence. Bcl-2, Bax, Cleaved Caspase-3, Caspase-3, CREB, P-CREB, ERK1/2, P-ERK1/2 and OTR were detected by western blotting.

### Cell Viability Assay

Hippocampal neurons were cultivated in 48-well plates for 7 d. Assay for cell viability refer to the literature (Chang et al., 1998). Hippocampal neurons cultured for 7 d were incubated with drugs for a certain period of time at 37°C, and four replicate wells were set in each group. Fresh culture medium and a final concentration of 0.25 mg/ml 3-(4,5-dimethylthiazol-2-yl)-2,5-diphenyl-tetrazolium bromide (MTT) solution were added during the test, and the cells were incubated in a 37°C incubator (95% air, 5% CO_2_) for 4 h. The medium was then aspirated, an equal volume of DMSO solvent was added and mixed with a micro-vibrator for 10 min. Absorbances were read at 570 nm using a microplate reader. The experiment was repeated for 6 times. Viable cells (cell viability, CV) were measured in percentage with respect to the following formula:

CV(%)=(OD (sample))/(OD (Control))×100%

### Detection of Reactive Oxygen Species in Neurons

Fluorescence intensity changes of Dichloro-dihydro-fluorescein diacetate (DCFH-DA) could quantitatively detect intracellular ROS levels. DCFH-DA itself has no fluorescence and could pass through the cell membrane freely. After entering the cell, it could be hydrolyzed by intracellular esterase to form DCFH. DCFH does not penetrate the cell membrane, making it easy for the probe to be loaded into the cell. DCFH could be converted to DCF after exposure to ROS. By measuring the fluorescence of DCF, the level of intracellular ROS could be known, and the fluorescence intensity is directly proportional to the intracellular ROS level. Rosup is a drug that raises intracellular ROS. Rosup (100 μM) was added to the untreated cell culture plates and incubated for 30 min in a 37°C incubator. The drug-treated medium was discarded, and fluorescent probe DCFH-DA (10 μM) was added to the drug-treated, Rosup-treated cells in a 37°C incubator for 20 min in the dark. The loaded cells were washed with serum-free medium to remove residual probes. Then an appropriate amount of cell culture medium was added to the cells, observed with a confocal microscope (Nikon, C2 Plus).

### Detection of Mitochondrial Membrane Potential

When the mitochondrial membrane potential is high, 5,5′,6,6′-Tetrachloro-1,1′,3,3′-tetraethyl-imidacarbocyanine iodide (JC-1) aggregates in the matrix of mitochondria in the form of polymer, which could produce red fluorescence; when the mitochondrial membrane potential was low, JC-1 could not accumulate in the matrix of mitochondria, only exist in the cytosol, JC-1 was a monomer at this time, which could produce green fluorescence. The relative proportion of commonly used red-green fluorescence was a measure of the proportion of mitochondrial depolarization. Decreased mitochondrial membrane potential was a hallmark feature of early apoptosis. CCCP (10 μM, a drug that lowers mitochondrial membrane potential) was added to untreated cells and incubated for 20 min in a 37°C incubator. The drug-treated and CCCP-treated hippocampal neurons were gently washed once with PBS and then mixed with the culture solution with JC-1 staining solution and the cells were incubated for 20 min in a 37°C incubator. After the incubation, the culture solution was aspirated and washed twice with pre-cooled JC-1 staining buffer. Then an appropriate amount of cell culture medium was added to the cells, observed with a confocal microscope (Nikon, C2 Plus).

### Western Blotting

The cells were lysed with an appropriate amount of ice-cold RIPA lysis buffer (97% RIPA strong lysate, 1% benzylsulfonyl fluoride, 1% NaF, 1% Na_3_VO_4_), then the cells were disrupted with a 1 ml syringe and allowed to stand at low temperature for 30 min. After the sample was cryopreserved, the BCA method was used to determine the total protein concentration, and then RIPA was used to adjust the total protein concentration of each group to the same concentration. Add loading buffer to each cell lysate, vortex and centrifuge to mix well, then heated at 95–100°C for 10 min to fully denature the protein. Proteins of each group of samples were separated using 10% SDS-polyacrylamide gels (SDS-PAGE). The separated proteins were transferred onto polyvinylidene fluoride membranes. After blocking in 5% skim milk, the membranes were incubated with primary antibodies including anti-Caspase-3 antibody (Cell Signaling, 9664T), anti-Cleaved Caspase-3 antibody (Cell Signaling, 9661S), Bax antibody (Cell Signaling, 2772T), CREB antibody (Cell Signaling, 9197S), Anti-Phospho-CREB (Cell Signaling, 9198S), Anti-ERK1/2 (Cell Signaling, 4695S), Anti-Phospho- ERK1/2 (Cell Signaling, 4370S), Anti-Oxytocin Receptor Antibody (Abcam, ab217212), BCL-2 Polyclonal Antibody (Proteintech, 12789-1-AP), Beta-actin Antibody (Santa Cruz Biotechnology, sc-47778), GAPDH Antibody (Hangzhou Xianzhi Biological Technology Co., Ltd., AB-MM 001). The membranes were incubated with horseradish peroxidase-conjugated secondary antibodies (1:5,000) at room temperature. Finally, place the membranes on the blackboard and add an appropriate amount of ECL illuminant to expose it on the imager (Bio-Tanon, Tanon-2500R). The experiments were replicated three times.

### Statistical Analysis

All data were statistically analyzed using SPSS 21.0 software. Western blot images and fluorescent images were processed using image J software and IPP 6.0 software. The data was plotted using GraphPad Prism 6.02 software. All values were expressed as means ± SEM. One-Way ANOVA was used to determine the overall significant difference between groups. The LSD test was used when the variance was equal, and the Dunnett-t test was used when the variance was not observed. A value of *p* < 0.05 was considered as statistically significant.

## Results

### Oxytocin Attenuates Methamphetamine-Induced Cytotoxic Effect in Hippocampal Neurons

**FIGURE 1:**
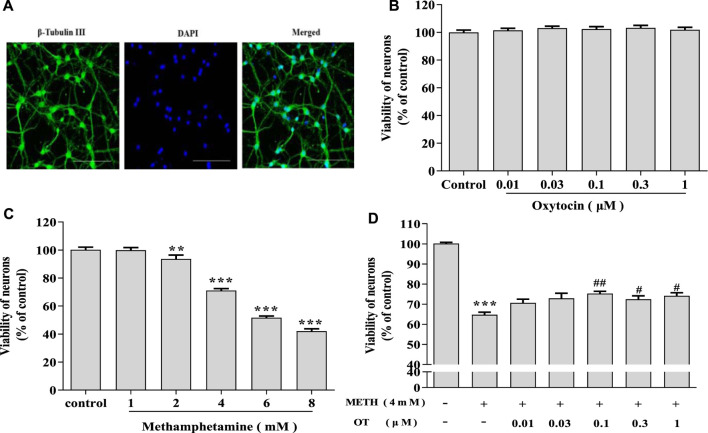
OT attenuates the decrease in METH-induced hippocampal neuron survival. **(A)** Immunochemical identify of hippocampal neurons by fluorescence dyeing. Neurons were stained with Cytoskeleton (green) (×20 objective, Scale bars = 50 μm). Nuclei were counter stained with DAPI (blue). **(B)** Hippocampal neurons were treated with OT (0.01–1 μM) for 24 h **(C)** Hippocampal neurons were treated with METH (1–8 mM) for 24 h. **(D)** Hippocampal neurons were pre-treated with OT (0.01–1 μM) for 2 h and stimulated with METH (4 mM) for 24 h before detected. All the values were shown as mean ± SEM (*n* = 3). **p < 0.01, ***p < 0.001 versus control groups; ^#^ *p* < 0.05, ^##^ *p* < 0.01 versus METH groups.

Primary hippocampal neurons cultured *in vitro* for 7 d were subjected to purity analyses using immunofluorescence staining with beta-Tubulin III antibody and DAPI. Purity of hippocampal neurons was 90% (Figure 1A), which could be used in subsequent experiments as described (Xu et al., 2014). As shown in Figure 1C, hippocampal neurons were treated with METH (1–8 mM) and the cell viability gradually decreased along with the increase of METH concentration. Compared with the control, 4 mM METH significantly reduced the viability of hippocampal neurons, which are similar to METH concentration reported in the literature (Qiao et al., 2014). Therefore, 4 mM METH was selected as the subsequent model concentration. MTT assay indicated that OT (0.01–1 μM) did not exhibit a toxic effect on the neurons (Figure 1B). Afterwards, the neurons were pretreated with OT (0.01–1 μM) for 2 h, followed by a 24 h’ stimulation with METH (4 mM). Figure 1D showed that OT (0.1–1 μM) significantly attenuated the decrease in the vitality of hippocampal neurons induced by METH.

### Oxytocin Receptor Activation Is Involved in the Prevention of Methamphetamine-Induced Cytotoxicity in Hippocampal Neurons

**FIGURE 2:**
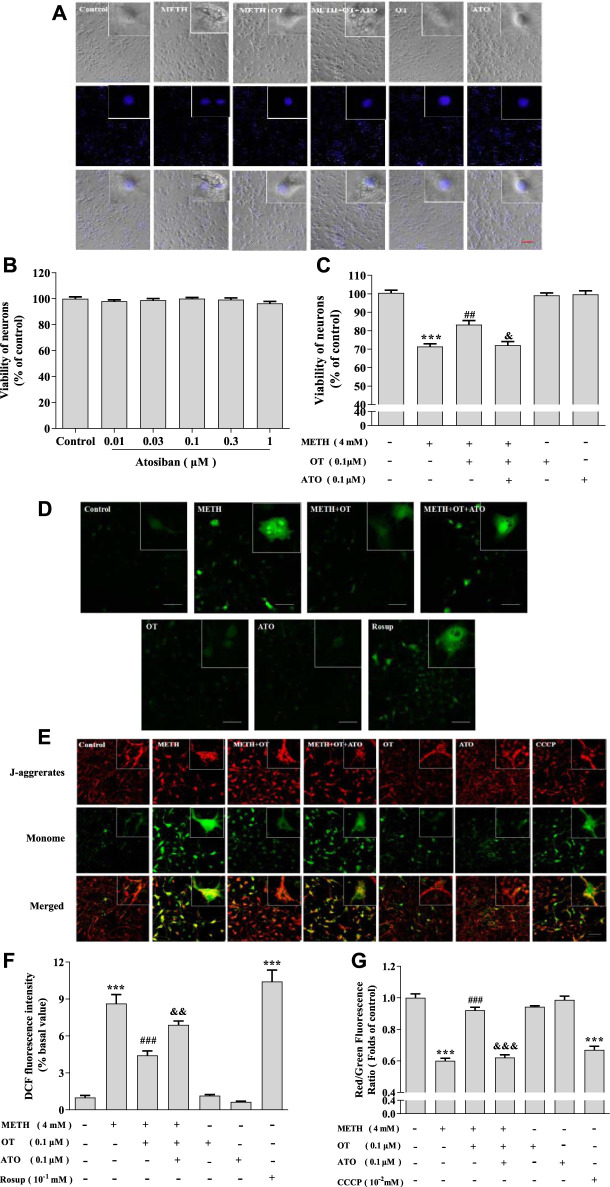
Oxytocin receptor participated in the prevention of METH -induced cytotoxicity in hippocampal neurons. **(A)** Hippocampal were pre-treated with OT and ATO for 2 h and stimulated with METH for 24 h. The cells were observed under a fluorescence microscope. (×20 objective; Scale bar = 50 μm). **(B)** Hippocampal neurons were treated with ATO (0.01–1 μM) for 24 h. **(C)** Hippocampal neurons were pre-treated with OT (0.1 μM) and ATO (0.1 μM) for 2 h and stimulated with METH (4 mM) for 24 h before detected. **(D,F)** Cells were observed by confocal microscope after stained with the Fluorescent dye DCFH-DA, DCF (Green fluorescence) measures ROS production. (×20 objective; Scale bar = 50 μm). **(E,G)** Cells were observed by confocal microscope after stained with the Fluorescent dye JC-1, JC-1 polymer/JC-I monomer (red/green fluorescence) measures the level of mitochondrial membrane potential. (×20 objective; Scale bar = 50 μm). The data were shown as mean ± SEM (*n* = 3). ***p < 0.001 compared with control groups; ^##^ *p* < 0.01, ^###^ *p* < 0.001 compared with METH groups; ^&^ *p* < 0.05, ^&&^ *p* < 0.01, ^&&&^ *p* < 0.001 compared with METH + OT groups.

It has been reported that ATO can antagonize the neuroprotective effect of OT on an equal dose (Ceanga et al., 2010; Kaneko et al., 2016). In order to clarify the role of oxytocin receptor, we carried out an experiment designed as follow: ATO (0.1 μM) and OT (0.1 μM) were pre-incubated for 2 h, and then incubated with METH (4 mM) for 24 h. MTT assay indicated that ATO (0.01–1 μM) did not show a toxic effect (Figure 2B). As shown in Figure 2A, OT could inhibit the enlargement of the cell body and the shrinkage of the nucleus induced by METH and this effect could be prevented by ATO. Similar results could also be obtained from MTT experiment (Figure 2C). We tested whether this effect was reflected in other cytotoxicity indicators of METH, including ROS levels and mitochondrial membrane potential levels. As result, OT pre-incubation prevented the decrease of mitochondrial membrane potential and the increased of ROS in hippocampal neurons induced by METH. Pre-incubation with ATO significantly prevented these changes (Figures 2D–G). These results indicate that oxytocin receptor participated in the prevention of METH-induced cytotoxicity in hippocampal neurons.

### Oxytocin Attenuates Methamphetamine-Induced Changes on Apoptotic Proteins in Hippocampal Neurons

**FIGURE 3:**
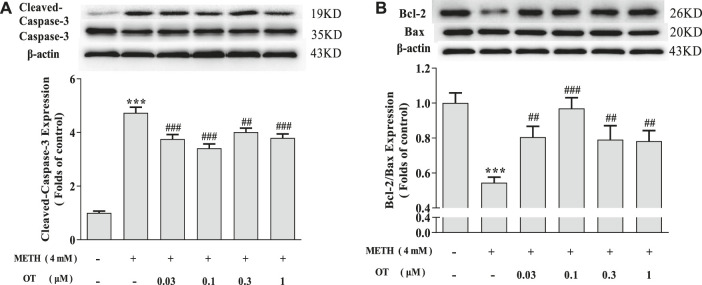
Effects of OT on the expression of apoptosis related proteins changed by METH in hippocampal neurons. **(A, B)** Hippocampal neurons were pre-treated with OT (0.03–1 μM) for 2 h and stimulated with METH (4 mM) for 24 h by western blotting. The data were shown as mean ± SEM (*n* = 3). ***p < 0.001 compared with control groups; ^##^ *p* < 0.01, ^###^ *p* < 0.001 compared with METH groups.

As shown in Figures 3A,B, METH increased the expression of Cleaved-Caspase-3 and decreased Bcl-2/Bax in hippocampal neurons. Pre-treatment with OT significantly attenuated the up-regulation of Cleaved-Caspase-3 expression and the down-regulation of Bcl-2/Bax expression induced by METH.

### Oxytocin Attenuates Methamphetamine-Induced Apoptosis *via* Oxytocin Receptor in Hippocampal Neurons

**FIGURE 4:**
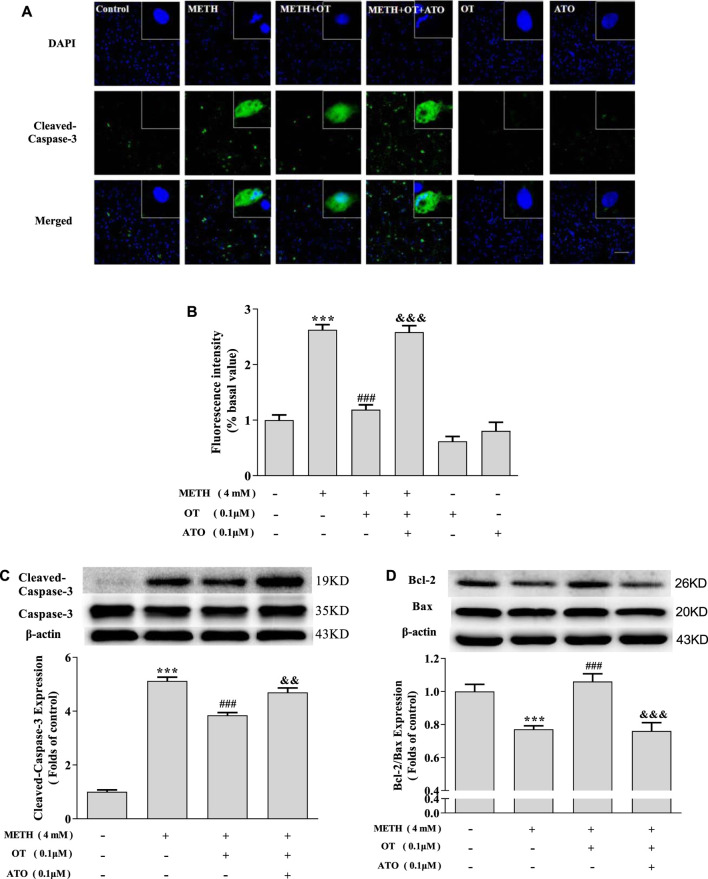
The effect of pre-incubation ATO and OT on METH-induced changes in the expression of Cleaved-Caspase-3 and Bcl-2/Bax. **(A, B)** Hippocampal neurons were pre-treated with OT (0.1 μM) and ATO (0.1 μM) for 2 h and stimulated with METH for 24 h. The panels in the columns marked “Merged” are merged images of the panels in the two left columns. (×20 objective; Scale bar = 50 μm). Each image by (IOD sum)/(area sum) represent the Cleaved-caspase -3 levels. **(C-D)** Hippocampal neurons were pre-treated with OT (0.1 μM) and ATO (0.1 μM) for 2 h and stimulated with METH (4 mM) for 24 h by western blotting. The data were shown as mean ± SEM (*n* = 3). ***p < 0.001 compared with control group; ^###^ *p* < 0.001 compared with METH group; ^&&^ *p* < 0.01, ^&&&^ *p* < 0.001 compared with METH + OT group.

As shown in Figures 4A–C, OT pre-incubation attenuated hippocampal neuronal nuclear shrinkage partial nuclear fragmentation and the up-regulation of Cleaved-Caspase-3 expression induced by METH. Pre-incubation with ATO significantly prevented these effects of OT. Similarly, Pre-incubation with ATO significantly reduced the expression of Bcl-2/Bax compared to METH + OT group (Figure 4D). These results indicate that OT could significantly attenuate METH-induced apoptosis *via* oxytocin receptor in hippocampal neurons.

### Oxytocin Prevents Methamphetamine-Induced Oxytocin Receptor Density Reduction in Hippocampal Neurons

**FIGURE 5:**
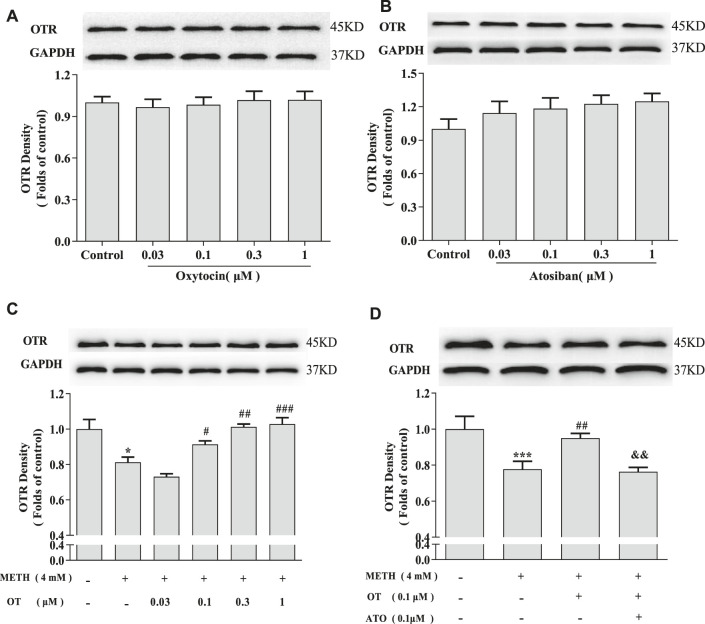
Effects of OT and ATO on the expression of oxytocin receptor changed by METH in the hippocampal neurons. **(A)** Hippocampal neurons were treated with OT (0.03–1 μM) for 24 h. **(B)** Hippocampal neurons were treated with ATO (0.03–1 μM) for 24 h. **(C)** Hippocampal neurons were pre-treated with OT (0.03–1 μM) for 2 h and stimulated with METH (4 mM) for 24 h by western blotting. **(D)** Hippocampal neurons were pre-treated with OT (0.1 μM) and ATO (0.1 μM) for 2 h and stimulated with METH (4 mM) for 24 h by western blotting. The data were shown as mean ± SEM (*n* = 3). *p < 0.05, ***p < 0.001 compared with control groups; ^#^ *p* < 0.05, ^##^ *p* < 0.01, ^###^ *p* < 0.001 compared with METH groups; ^&&^ *p* < 0.01 compared with METH + OT groups. Oxytocin Attenuates the Decrease of Phospho-CREB Expression Induced by Methamphetamine Through Oxytocin Receptor in Hippocampal Neurons.

In order to explore how OT influences on oxytocin receptor in the injured hippocampal neurons, we investigated the changes of the density of oxytocin receptor *via* western blotting. As shown in Figures 5A,B, OT and ATO had no significant effect on the density of oxytocin receptor in hippocampal neurons in our experiment condition. Figures 5C,D showed that METH (4 mM) significantly decreased the density of oxytocin receptor. Pre-incubation with OT (0.1–1 μM) prevented the decrease of oxytocin receptor density induced by METH. Pre-incubation with ATO significantly prevented this effect.

**FIGURE 6:**
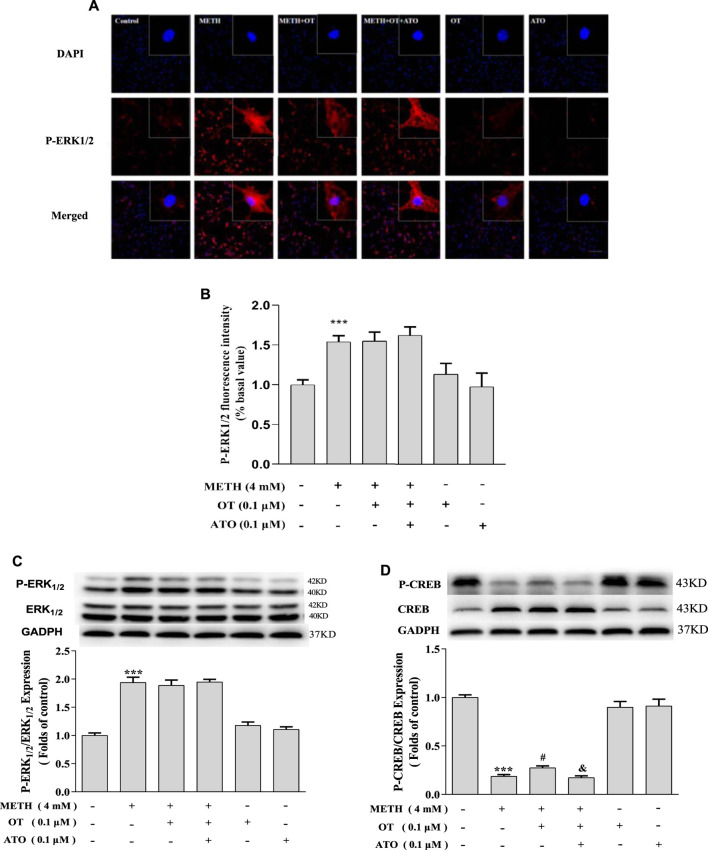
Effects of OT and ATO on the expression of P-ERK1/2 and P-CREB changed by METH in the hippocampal neurons. **(A–D)** Hippocampal neurons were pre-treated with OT (0.1 μM) and ATO (0.1 μM) for 2 h and stimulated with METH (4 mM) for 24 h by fluorescence dyeing and western blotting. The data were shown as mean ± SEM (*n* = 3). ***p < 0.001 compared with control groups; ^#^ *p* < 0.05 compared with METH groups; ^&^ *p* < 0.05 compared with METH + OT groups.

METH significantly increased the expression of p-ERK1/2 in hippocampal neurons compared with the control group, whereas pre-administration of OT and ATO could not attenuate the increase of p-ERK1/2 expression induced by METH (Figures 6B,C). Pre-treatment with OT significantly attenuated the decrease of p-CREB induced by METH (Figure 6D). Pre-treatment with ATO could prevent this effect (Figure 6D). These results indicate that OT could attenuate METH-induced p-CREB expression reduction through the oxytocin receptor in hippocampal neurons.

## Discussion

Studies have shown that METH could cause DNA strand breaks, chromatin condensation and nuclear lysis, indicating that METH could induce apoptosis (Cadet et al., 1997). Previously studies have demonstrated that OT has inhibitory effects against METH-induced conditioned place preference (CPP) and the recurrence of CPP caused by restraint stress, which could be prevented by ATO (Qi et al., 2009). However, there are few researches on the protective mechanism of OT on METH-induced apoptosis in hippocampal neurons. Therefore, we hypothesized that if oxytocin receptors are expressed in hippocampal neurons, OT may inhibit or attenuate METH-induced apoptosis by activating oxytocin receptor. As expected, our results indicate that oxytocin receptor activation is involved in the preventive effect of OT on METH-induced apoptosis in rat hippocampal neurons.

The hippocampus is part of the most studied in the central nervous system of mammals. Changes in the structure and function of the hippocampus have been demonstrated to play an extremely important role in many diseases. Primary cultured hippocampal neurons can reflect their state *in vivo* to a certain extent and have been widely used in neuroscience study. The cytotoxicity of METH could lead to apoptosis, and a decrease in cell viability can be measured *in vitro*. MTT results showed that 4 mM METH could significantly reduce the viability of hippocampal neurons. Literature has shown that *in vitro* experiments, 3 mM METH could damage cells through a process involving DNA fragmentation between nucleosomes and chromatin condensation, which is consistent with the process of apoptosis (Cadet et al., 1997; Qiao et al., 2014). It shows that *in vitro* experiments, acute treatment of cells with high doses of METH could induce cell apoptosis, and cell death is not caused by the non-specific toxicity of the drug. MTT and morphological results showed that ATO could prevent the protective effect of OT on hippocampal neurons. It shows that OT could attenuate the decreased vitality of hippocampal neurons caused by METH through oxytocin receptor. On the other hand, the decrease of mitochondrial membrane potential and ROS have been shown to play an import role in METH-induced toxicity (Ramkissoon and Wells, 2015). OT and other antioxidants can resist the decrease of mitochondrial membrane potential induced by METH (Wagner et al., 1985; De Vito and Wagner, 1989; Wu et al., 2006). For the above, we next examined ROS levels and the changes in mitochondrial membrane potential in each group of cells. Consistent with our expectations, we confirmed that OT could prevent the decrease of mitochondrial membrane potential and the increase of ROS induced by METH in hippocampal neurons. But all these effects could be prevented by ATO.

METH promotes the release of glutamate after entering neurons, and activates NMDA receptor to increase the production of ROS and nitric oxide. In addition, METH enters the mitochondria to increase the pH of the inner membrane matrix, thereby reducing the activity of ATP synthase, resulting in a decrease in the ability of mitochondria to maintain membrane potential. At the same time, it promotes the release of large amounts of Ca^2+^ from the mitochondrial permeability transition pore, activates nitric oxide, reduces the amount of Bcl-2, and activates the caspase cascade to induce apoptosis (Davidson et al., 2001). Previous studies have shown that OT could inhibit the decrease of Bcl-2 and the increase of Bax in the striatum induced by rotenone (Taylor et al., 2008). Bcl-2/Bax was usually used to indicate mitochondrial permeability of damaged cells (Li et al., 2003; Newhouse et al., 2004). Western blotting results showed that METH significantly decreased the expression of Bcl-2 but did not alter the expression of Bax protein. We speculated that Bax protein expression is different due to the presence of drug concentration, cell type and duration of action. Despite this, METH significantly decreased the expression of Bcl-2/Bax and increased the expression of Cleaved-Caspase-3, indicating that METH could promote hippocampal neuronal apoptosis. Pre-treatment with OT significantly attenuated the pro-apoptotic effect of METH. Similar to MTT assay, ATO could prevent this effect of OT. These results further demonstrated that OT can exert an anti-apoptotic effect through oxytocin receptor.

To investigate how OT exerts neuroprotective effects through oxytocin receptor, we first studied the density of oxytocin receptor in each group. Our results show that OT and ATO had no significant effects on the density of oxytocin receptor in hippocampal neurons. Additionally, METH significantly reduced the density of oxytocin receptor. OT pre-treatment prevented METH-induced reduction of oxytocin receptor density in rat hippocampal neurons, and pre-treatment with ATO prevented this effect. *In vivo* studies have shown that chronic intraperitoneal injection of METH in male CD1 mice can cause an up-regulation of oxytocin receptor density in specific areas of the amygdala and hypothalamus, but not in the nucleus accumbens and caudate shell. These changes may be the result of long-term use of METH caused by the neurological adaptation of oxytocin receptor system in related brain areas, but the significance of the changes in oxytocin receptor in these brain areas is not fully understood (Zanos et al., 2014). Our results indicate that METH also caused changes on oxytocin receptor in rat hippocampal neurons cultured *in vitro*, and OT could prevent these changes. This may be related to part of the behavior of OT in the treatment of METH addiction in rodents in vivo studies. In fact, studies have shown that the oxytocin receptor mediates the inhibitory effect of OT on the recurrence of conditioned place preference caused by METH-induced conditioned place preference and restraint pressure (Qi et al., 2009). At least our results show that OT treatment is beneficial to METH-injured neurons, and provides certain *in vitro* theoretical support for OT treatment of METH-induced nerve damage. At the same time, in order to study whether OT participates in certain cellular pathways through oxytocin receptor to affect the apoptosis caused by METH in rat hippocampal neurons. We also explored whether the ERK1/2-CREB pathway is involved in the neuroprotective effects of OT.

ERK1/2 is the first-found MAPKs protein kinase in mammals (Cooper et al., 1982). In most mammalian MAPKs cascade, ERK1/2 pathway is mainly activated by stress stimuli and growth factors. ERK1/2 pathway is generally involved in cell survival (Giancotti, 1997). However, depending on the type of stimulus, ERK1/2 has also been reported to be associated with cell death through apoptosis and a second type of programmed cellular mechanism (Ulivi et al., 2010). Some studies have found that the neurotoxicity caused by carmustine is partly caused by activation of ERK1/2, and Chlorpyrifos can activate ERK1/2 through ROS to cause nerve cell death, indicating that ERK1/2 activation can cause cytotoxic damage (An et al., 2011; Lee et al., 2012). ERK1/2 also have the potential to resist neurodegeneration by inhibiting caspase and the apoptotic pathway (Karmarkar et al., 2011). ERK1/2 activation could remarkably increase the phosphorylation of CREB, the elevated phosphorylation of CREB contributes to the activation of Bcl-2, which functions as an anti-apoptotic protein, consequently preventing neuronal death (Meller et al., 2005). Similar to the previous results, OT attenuated the up-regulation of P-CREB expression induced by METH *via* oxytocin receptor. However, our results suggested that OT did not attenuate METH-induced increase in P-ERK1/2 expression. It has been reported in the literature that other MAPKs signaling pathways such as P38 and JNK pathways are involved in methamphetamine-induced apoptosis. The use of P38 inhibitors could significantly attenuate the apoptosis of hippocampal neurons induced by METH (Zhu, et al., 2018). The use of JNK inhibitor 600,125 attenuated the METH-induced apoptosis in SH-SY5Y cells (Wang, et al., 2008). In this study, the specific mechanism needs to be further explored.

In conclusion, our study proved that OT could significantly attenuate METH-induced apoptosis in rat hippocampal neurons and this protective effect involves activation of oxytocin receptor. Our results provide some theoretical basis for the protection of OT against neuronal damage induced by METH abuse.

## Data Availability

The original contributions presented in the study are included in the article/Supplementary Files, further inquiries can be directed to the corresponding authors.
