# Supplementary figures and images for: Oxytocin Attenuates Methamphetamine-Induced Apoptosis via Oxytocin Receptor in Rat Hippocampal Neurons
Source: Front Pharmacol. 2021 Aug 12;12:639571. doi: 10.3389/fphar.2021.639571 (PMC8415150; doi:10.3389/fphar.2021.639571)

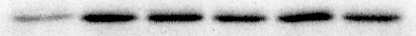

Supplement: Supplementary file 1 [file DataSheet1.ZIP › ╨┬╜¿╬─╝■╝╨/Fig.3/Fig.3A c-cas-3.jpg]

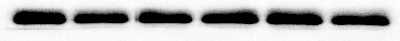

Supplement: Supplementary file 1 [file DataSheet1.ZIP › ╨┬╜¿╬─╝■╝╨/Fig.3/Fig.3A cas-3...jpg]

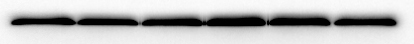

Supplement: Supplementary file 1 [file DataSheet1.ZIP › ╨┬╜¿╬─╝■╝╨/Fig.3/Fig.3A a┬-actin.jpg]

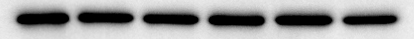

Supplement: Supplementary file 1 [file DataSheet1.ZIP › ╨┬╜¿╬─╝■╝╨/Fig.3/Fig.3B Bax.jpg]

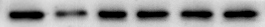

Supplement: Supplementary file 1 [file DataSheet1.ZIP › ╨┬╜¿╬─╝■╝╨/Fig.3/Fig.3B Bcl-2.jpg]

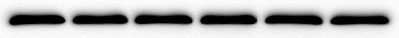

Supplement: Supplementary file 1 [file DataSheet1.ZIP › ╨┬╜¿╬─╝■╝╨/Fig.3/Fig.3B a┬-actin.jpg]

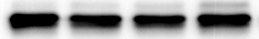

Supplement: Supplementary file 1 [file DataSheet1.ZIP › ╨┬╜¿╬─╝■╝╨/Fig.4/Fig.4C caspase-3.jpg]

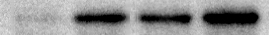

Supplement: Supplementary file 1 [file DataSheet1.ZIP › ╨┬╜¿╬─╝■╝╨/Fig.4/Fig.4C cle-cas-3.jpg]

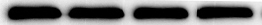

Supplement: Supplementary file 1 [file DataSheet1.ZIP › ╨┬╜¿╬─╝■╝╨/Fig.4/Fig.4C a┬-actin.jpg]

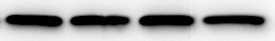

Supplement: Supplementary file 1 [file DataSheet1.ZIP › ╨┬╜¿╬─╝■╝╨/Fig.4/Fig.4D Bax.jpg]

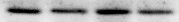

Supplement: Supplementary file 1 [file DataSheet1.ZIP › ╨┬╜¿╬─╝■╝╨/Fig.4/Fig.4D Bcl-2.jpg]

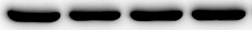

Supplement: Supplementary file 1 [file DataSheet1.ZIP › ╨┬╜¿╬─╝■╝╨/Fig.4/Fig.4D a┬-actin.jpg]

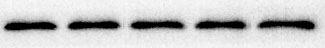

Supplement: Supplementary file 1 [file DataSheet1.ZIP › ╨┬╜¿╬─╝■╝╨/Fig.5/Fig.5A GAPDH.jpg]

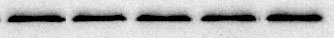

Supplement: Supplementary file 1 [file DataSheet1.ZIP › ╨┬╜¿╬─╝■╝╨/Fig.5/Fig.5A OTR.jpg]

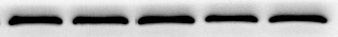

Supplement: Supplementary file 1 [file DataSheet1.ZIP › ╨┬╜¿╬─╝■╝╨/Fig.5/Fig.5B GAPDH.jpg]

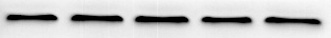

Supplement: Supplementary file 1 [file DataSheet1.ZIP › ╨┬╜¿╬─╝■╝╨/Fig.5/Fig.5B OTR.jpg]

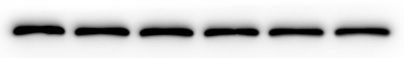

Supplement: Supplementary file 1 [file DataSheet1.ZIP › ╨┬╜¿╬─╝■╝╨/Fig.5/Fig.5C GAPDH.jpg]

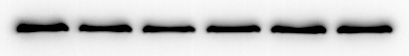

Supplement: Supplementary file 1 [file DataSheet1.ZIP › ╨┬╜¿╬─╝■╝╨/Fig.5/Fig.5C OTR.jpg]

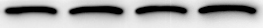

Supplement: Supplementary file 1 [file DataSheet1.ZIP › ╨┬╜¿╬─╝■╝╨/Fig.5/Fig.5D GAPDH.jpg]

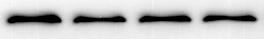

Supplement: Supplementary file 1 [file DataSheet1.ZIP › ╨┬╜¿╬─╝■╝╨/Fig.5/Fig.5D OTR.jpg]

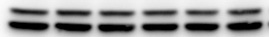

Supplement: Supplementary file 1 [file DataSheet1.ZIP › ╨┬╜¿╬─╝■╝╨/Fig.6/Fig.6B ERK.jpg]

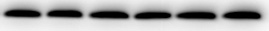

Supplement: Supplementary file 1 [file DataSheet1.ZIP › ╨┬╜¿╬─╝■╝╨/Fig.6/Fig.6B GAPDH.jpg]

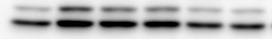

Supplement: Supplementary file 1 [file DataSheet1.ZIP › ╨┬╜¿╬─╝■╝╨/Fig.6/Fig.6B P-ERK.jpg]

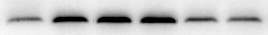

Supplement: Supplementary file 1 [file DataSheet1.ZIP › ╨┬╜¿╬─╝■╝╨/Fig.6/Fig.6C CREB.jpg]

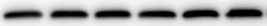

Supplement: Supplementary file 1 [file DataSheet1.ZIP › ╨┬╜¿╬─╝■╝╨/Fig.6/Fig.6C GAPDH.jpg]

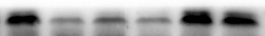

Supplement: Supplementary file 1 [file DataSheet1.ZIP › ╨┬╜¿╬─╝■╝╨/Fig.6/Fig.6C P-CREB.jpg]
